# Is the Hippo Pathway Effector Yes-Associated Protein a Potential Key Player of Dairy Cattle Cystic Ovarian Disease Pathogenesis?

**DOI:** 10.3390/ani13182851

**Published:** 2023-09-08

**Authors:** Esdras Corrêa Dos Santos, Alexandre Boyer, Guillaume St-Jean, Natalia Jakuc, Nicolas Gévry, Christopher A. Price, Gustavo Zamberlam

**Affiliations:** 1 Centre de Recherche en Reproduction et Fertilité (CRRF), Faculté de Médecine Vétérinaire (FMV), Université de Montréal (UdeM), Saint-Hyacinthe, QC J2S 7C6, Canada; 2 Département de Biomédecine Vétérinaire, Faculté de Médecine Vétérinaire (FMV), Université de Montréal (UdeM), Saint-Hyacinthe, QC J2S 7C6, Canada; 3 Département de Pathologie et Microbiologie, Faculté de Médecine Vétérinaire (FMV), Université de Montréal (UdeM), Saint-Hyacinthe, QC J2S 7C6, Canada; 4 Département de Biologie, Faculté des Sciences, Université de Sherbrooke, Sherbrooke, QC J1K 0A5, Canada

**Keywords:** ovary, cow, Hippo, CTGF, CYR61, ANKRD1, TEAD, anovulation, cyst, COD, granulosa cells, theca cells

## Abstract

**Simple Summary:**

Cystic ovarian disease is an important ovarian disorder that leads to anovulatory infertility in dairy cows. In the present study, we used spontaneous ovarian follicular cysts to show the first evidence that the deregulation of Hippo pathway effector yes-associated protein expression and/or activity can be a potential key to better understand cystic ovarian disease pathogenesis. This finding also allows us to point towards Hippo pathway as a potential therapeutic target for the treatment of this major ovarian disorder in cattle.

**Abstract:**

Cystic ovarian disease (COD) in dairy cattle is characterized by preovulatory follicles that become cysts, fail to ovulate and persist in the ovary; consequently, interfering with normal ovarian cyclicity. The intraovarian key players that orchestrate the alterations occurring in the preovulatory follicle and that culminate with cyst formation and persistence, however, remain uncertain. Interestingly, the Hippo pathway effector yes-associated protein (YAP) has been described in humans and mice as a key player of anovulatory cystic disorders. To start elucidating if YAP deregulation in ovarian follicle cells can be also involved in the pathogenesis of COD, we have generated a series of novel results using spontaneously occurring cystic follicles in cattle. We found that mRNA and protein levels of YAP are significantly higher in granulosa (GCs) and theca cells (TCs) isolated from cystic follicles (follicular structures of at least 20 mm in diameter) in comparison to respective cell types isolated from non-cystic large follicles (≥12 mm). In addition, immunohistochemistry and Western blot analyses used to determine YAP phosphorylation pattern suggest that YAP transcriptional activity is augmented is cystic GCs. These results were confirmed by a significant increase in the mRNA levels encoding for the classic YAP-TEAD transcriptional target genes *CTGF*, *BIRC5* and *ANKRD1* in GCs from follicle cysts in comparison to non-cystic large follicles. Taken together, these results provide considerable insight of a completely novel signaling pathway that seems to play an important role in ovarian cystic disease pathogenesis in dairy cattle.

## 1. Introduction

The fertility of high-yielding dairy cows has been declining for decades due to many factors, and notably ovarian malfunctions are at the top of the list [1,2]. Among ovarian disorders observed in dairy cows, cystic ovarian disease (COD) is very common. COD has been defined by the presence (in one or both ovaries) of one or more follicular cysts larger than 20 mm in diameter and that persist for up to 10 days without luteal tissue [3]. This disorder directly causes reproductive failure and represents severe economic loss to the dairy industry because it increases both the days open in the post-partum period and replacement rates due to infertility [3,4].

Ovarian cysts normally develop from preovulatory follicles that have failed to ovulate, they persist in the ovary and thus interfere with normal ovarian cyclicity causing anovulatory infertility [5]. Although it has been accepted that the development of ovarian cysts is associated with an endocrine imbalance in the hypothalamic–pituitary–gonadal axis [6,7], the key intraovarian components involved in the mechanism of cyst formation and persistence remain uncertain. The Hippo pathway effector yes-associated protein (YAP) has emerged as a potential candidate.

The Hippo intracellular signaling pathway is evolutionarily highly conserved with well-known roles in cell differentiation, proliferation and apoptosis in several tissues, particularly during embryogenesis [8,9]. The core Hippo pathway consists of a kinase cascade that regulates the activity of the transcriptional activators YAP and transcriptional co-activator with PDZ-binding motif (TAZ). When phosphorylated, YAP/TAZ are sequestered in the cytoplasm or degraded, and are unable to alter gene transcription. Conversely, unphosphorylated YAP/TAZ accumulate in the nucleus and form complexes with distinct transcription factors, particularly those of the TEAD family, resulting in the regulation of the transcriptional activity of target genes in a cell type- and context-specific manner [9,10,11,12,13].

A number of studies indicate the importance of Hippo effectors to ovarian physiology in adult animals, including cattle [14,15,16]. Interestingly, it has been shown that overexpression of YAP negatively impacts LH action in mouse granulosa cells (GCs) [17] and that YAP is considered a susceptibility gene for polycystic ovarian syndrome in women [18,19]. It has been recently demonstrated that YAP transcriptional activity is necessary for activation of the LH-induced preovulatory cascade in bovine GCs, and that inhibiting YAP activity blocks ovulation in cattle in vivo [15]. However, it is unknown if YAP activity deregulation in the preovulatory follicle contributes to ovarian cyst formation in this species. To address this, the objectives of the present study were to measure activation of the main Hippo signaling pathway effector and the expression of classic YAP-TEAD target genes in spontaneously occurring cystic follicles.

## 2. Material and Methods

The reagents used in the present study were obtained from Thermo Fisher Scientific (Saint-Laurent, QC, Canada) except where otherwise stated.

### 2.1. Cystic Follicle Collection

Ovarian follicle cysts were selected based on macroscopic evaluation, and were identified as follicles larger than 20 mm and with a follicle wall of up to 3 mm thickness [20]; follicles with greater than 3 mm wall thickness were likely luteal cysts and were discarded. Histology was performed on a subset of the selected ovaries which were examined for the presence of classic alterations that are normally observed in cystic follicles, including partial loss of GCs with pyknotic cells, and interruption in the basement membrane with invasion of GCs into the hypertrophied theca cell layer [3,5,20]. Cysts presenting only a fibrous tissue capsule and liquid were not included in the present study. Non-cystic follicles were defined as those between 12 and 17 mm diameter.

Bovine ovaries were collected at different days from random adult cows at a local abattoir and were transported to the laboratory in PBS at 35 °C containing penicillin (100 IU/mL), streptomycin (100 μg/mL) and fungizone (1 μg/mL). For histology, ovaries were fixed in formalin (10% buffered), paraffin-embedded, stained in H&E and evaluated histologically. An approximate volume of 400 µL of follicular fluid was collected from each follicle and stored at −80 °C for hormone assay. GCs were then collected from the cystic and non-cystic follicles by aspiration and washed twice by centrifugation at 219× *g* for 10 min each. After assessing GC viability (with 0.4% Trypan blue stain) and counting the number of viable cells, 1 × 10^6^ viable cells were placed in RLT buffer for RNA extraction or in M-PER^®^ mammalian protein extraction reagent for protein extraction, and stored at −80 °C. Following GCs removal, some follicles were then used for TCs isolation. Briefly, the TCs layer was dissected from the surrounding stroma and washed several times with PBS plus heparin-enriched solution to remove all remaining attached GCs. Then, samples were aliquoted (50 mg each) for total RNA and protein extraction as described above. 

### 2.2. Immunohistochemistry

For immunohistochemistry (IHC) analyses, bovine ovaries and sample selection for experimental or control groups were performed as described above. At the laboratory, entire ovaries (at least four per group) were fixed in 10% formaldehyde solution for 24 h, rinsed and dehydrated in alcohol until embedded in paraffin. Serial sections were prepared (at a thickness of 3 µm) followed by deparaffinization, rehydration, sodium citrate heat-mediated antigen retrieval, peroxidase block and protein blocking (10% goat for 30 min), and then slides were probed with primary antibody against total and phosphorylated forms of YAP or against Pan-TEAD (Table 1) overnight at 4 °C. Protein detection was then performed with the Vectastain Elite ABC HRP Kit (VECTPK6101; Vector Laboratories Inc., Burlingame, CA, United States) and stained with DAB substrate kit (VECTSK4100; Vector Laboratories Inc., Burlingame, CA, United States). Slides were then counterstained with hematoxylin and dehydrated with graded alcohols prior to mounting. Negative controls consisted of slides for which the primary antibody was omitted. Photomicrographs were taken using a Carl Zeiss Axio Imager M1 microscope (Carl Zeiss Canada Ltd., Toronto, ON, Canada) at ×1000 magnification and using the Zen 2012 blue edition software (Carl Zeiss, Oberkochen, Germany).

### 2.3. Total RNA Extraction and Real-Time qPCR

Total RNA from GCs and TCs from control and cystic follicles were extracted using the Total RNA Mini Kit (Blood/Cultured Cell; Geneaid Biotech Ltd., New Taipei City, Taiwan) according to the manufacturer’s protocol. Reverse transcription was performed using 50 ng of total RNA and the SuperScript Vilo cDNA synthesis kit. Real-time qPCR was performed using Advanced supergreen qPCR mastermix (WISENT Inc., Saint-Bruno, QC, Canada) and a CFX96 Touch™ instrument (Bio-Rad, Hercules, CA, USA). Each PCR reaction consisted of 7.5 μL of SsoAdvanced SYBR Green PCR Master Mix, 2.3 μL of water, 4 μL of cDNA sample and 0.6 μL (10 pmol) of gene-specific primers (Table 2). Cycling conditions were 3 min at 95 °C followed by 40 cycles of 15 s at 95 °C, 30 s at 60 °C, and 30 s at 72 °C. In each run, melting curve analysis was used to verify that a single product was amplified. Each reaction was performed in duplicate and the average threshold cycle (Ct) value was used to calculate relative mRNA abundance of target genes relative to the geometric mean of three housekeeping genes (*H2AFZ*, *GAPDH* and *RPL19*) with the 2^−∆∆Ct^ method and correction for amplification efficiency [21]. Primers not published previously were designed based on sequences from GenBank, using Primer-BLAST platform and their respective amplicons were sequenced to confirm specificity.

### 2.4. Western Blotting

Total protein from GCs and TCs were extracted using M-PER^®^ mammalian protein extraction reagent according to the manufacturer’s instructions and protein levels were quantified using the Pierce™ BCA Protein Assay Kit. Halt™ Protease and Phosphatase Inhibitor Cocktails were added to the samples final solution to avoid protein degradation. Samples (20–40 μg) were resolved on 12% sodium dodecyl sulfate-polyacrylamide gels and transferred to Hybond-P PVDF membrane (GE Amersham, Amersham, UK). Membranes were then probed at 4 °C overnight in 5% BSA in TTBS with different primary antibodies (details and dilutions for each antibody are indicated in Table 1). After washing three times with TTBS, membranes were incubated for 1 h at room temperature with anti-rabbit HRP-conjugated IgG diluted in 5% non-fat dry milk in TTBS. Protein bands were visualized by chemiluminescence (ECL; Millipore, Burlington, MA, USA) and quantified using a ChemiDoc MP detection system (Bio-Rad) and Image Lab™ software version 6.0.1.

### 2.5. Hormone Assay

Follicular fluid concentrations of estradiol (E2), progesterone (P4) and testosterone (T) were measured by liquid chromatography–tandem triple quadrupole mass spectrometry (LC-MS/MS) in the Endocrine Technologies Core (ETC) at the Oregon National Primate Research Center (ONPRC) as previously described [25]. Briefly, 10 µL of follicular fluid was combined with deuterium-labeled standards for E2, P4 and T and subjected to supported liquid extraction (Biotage). Samples were then analyzed for hormone concentrations on a Shimadzu Nexera-LCMS-8050 (Shimadzu Scientific Instruments, Durham, NC, USA) LC-MS/MS instrument. Intra-assay coefficients of variation (CV) for E2, P4 and T were 2.8%, 6.0% and 1.4%, respectively, and inter-assay CVs for E2, P4 and T were 3.5%, 6.2% and 1.6%, respectively. 

### 2.6. Statistical Analysis

All analyses were performed using four to ten independent follicle samples per group (cystic or non-cystic), each isolated from ovaries collected at different days. The statistical analyses for all assessments were performed using GraphPad Prism software version 9.3.1 (GraphPad Software Inc., La Jolla, CA, USA). Data from mRNA abundance or target protein levels that were not normally distributed (Shapiro–Wilk test) were transformed to natural logarithms. Two-tailed *t*-tests were used to compare the two experimental groups. All data are presented as means ± SEM and variables that were considered statistically significant at *p* < 0.05 are represented with an asterisk symbol (*).

## 3. Results

### 3.1. Molecular and Hormonal Validation of COD Follicle Samples

**Figure 1:**
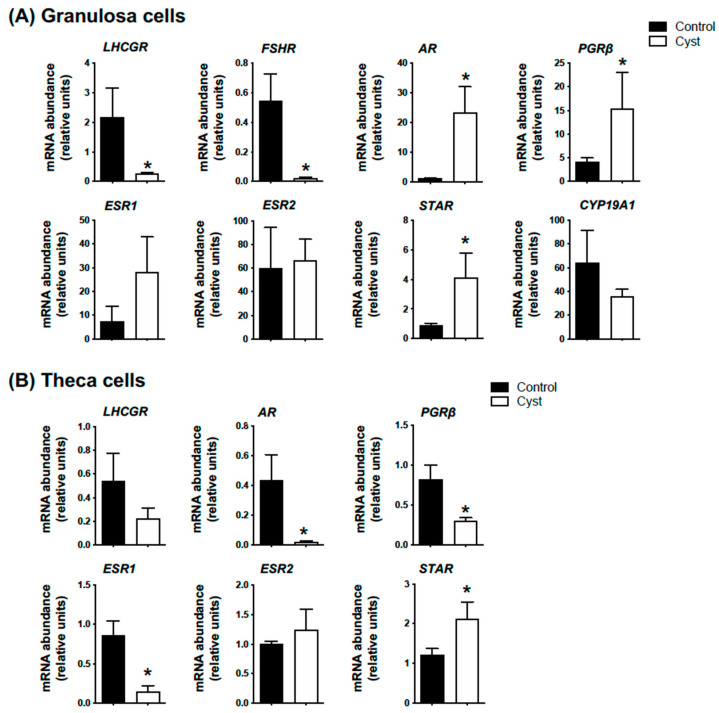
**Molecular validation of spontaneous cystic follicle samples.** Relative messenger RNA abundance was measured by real-time qPCR and normalized to the geometric mean of three housekeeping genes *H2AFZ*, *GAPDH* and *RLP19*. Granulosa (**A**) and theca cells (**B**) were isolated from ovarian follicular cysts (structures of at least 20 mm in diameter; Cyst group) and from non-cystic large follicles (≥12 mm; Control group). Data represent the mean ± SEM from granulosa (n = 10) and theca cells (n = 4) independent follicle samples per group. An asterisk (*) indicates significant difference between groups (*p* < 0.05).

We first confirmed if the spontaneously occurring cystic follicles collected from the abattoir presented the same expression pattern expected for classic markers for COD such as progesterone receptor beta (*PGRb*), androgen receptor (*AR*), steroidogenic acute regulatory protein (*STAR*), luteinizing hormone (*LHCGR*), follicle stimulating hormone receptor (*FSHR*) and estrogen receptors type 1 (*ESR1*) and 2 (*ESR2*). Abundance of mRNA encoding *PGRb*, *AR* and *STAR* was significantly increased in GCs from cysts compared to control GCs, and mRNA levels for both *LHCGR* and *FSHR* were significantly lower in cyst CGs in comparison to controls (*p* < 0.05; Figure 1A). Although a clear tendency was observed, neither *ESR1* nor *CYP19A1* mRNA abundance was statistically altered in cysts (*p* > 0.05; Figure 1A). In terms of theca cells gene expression profile, while *STAR* mRNA abundance was significantly increased (*p* < 0.05; Figure 1B), *PGRb*, *AR* and *ESR1* mRNA levels were significantly lower in cystic compared to control follicles (*p* < 0.05; Figure 1B). 

**Figure 2:**
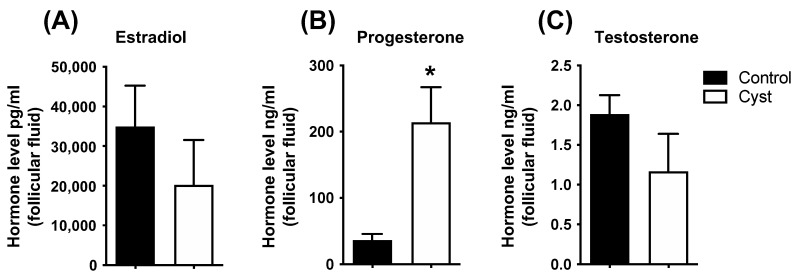
**Steroids concentration in follicular fluid of control and cyst groups.** Follicular fluid steroid concentrations for estradiol (**A**), progesterone (**B**) and testosterone (**C**) were measured by liquid chromatography–tandem triple quadrupole mass spectrometry. Data represent the mean ± SEM of independent follicle samples (n = 10) per group. An asterisk (*) indicates significant difference between groups (*p* < 0.05).

Progesterone concentrations in follicular fluid were significantly increased in cyst samples in comparison to controls (*p* < 0.05; Figure 2), whereas E2 and T concentrations were not statistically different between control and cyst groups (*p* > 0.05; Figure 2). Together, these results allowed us to confirm the COD status of the experimental samples.

### 3.2. YAP and Phospho-YAP Expression Pattern in Bovine Cystic Follicles

**Figure 3:**
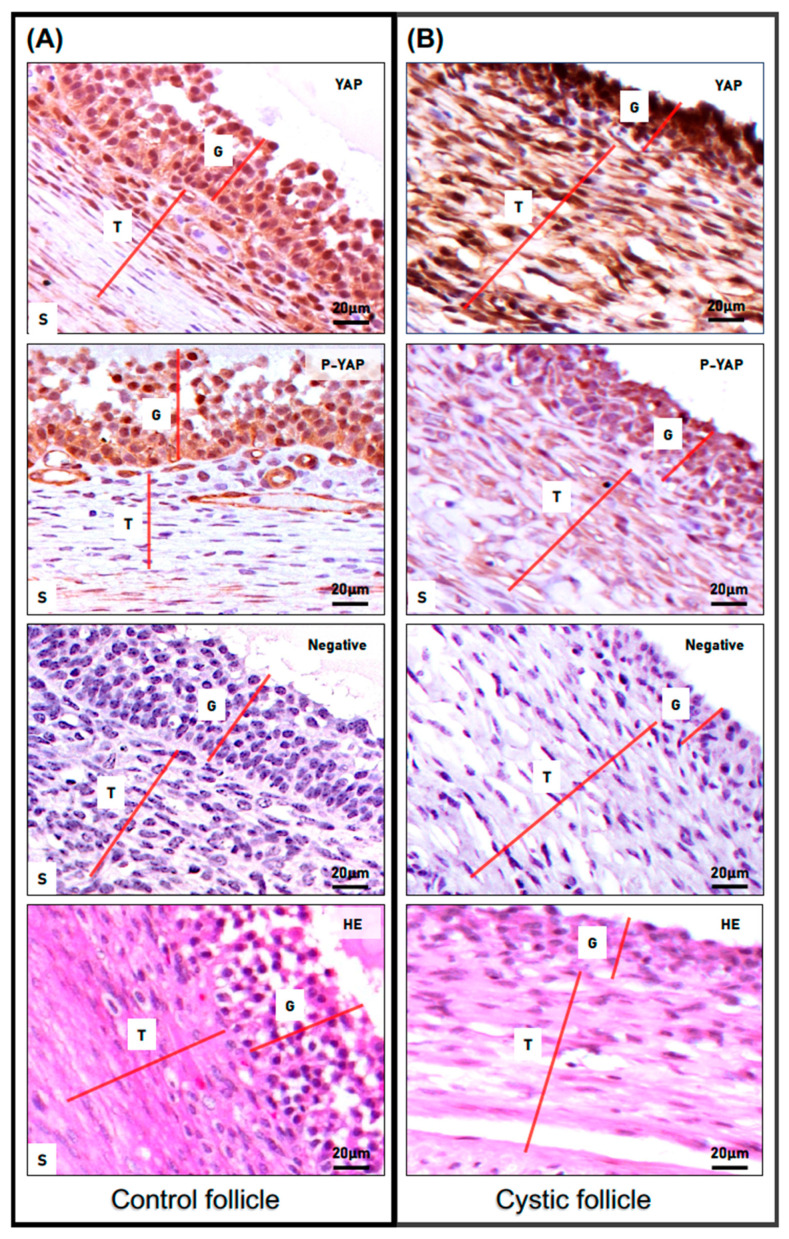
**Expression pattern of total and phosphorylated YAP proteins in spontaneous cystic follicles in cows.** Immunohistochemistry (IHC) analysis was used to compare the cellular and subcellular localization of total and phospho-YAP (P-YAP; Ser 127) proteins in control non-cystic large follicles (**A**) and in spontaneous cystic follicles (**B**). The red lines differentiate the distinct cell layers observed in the images (G: granulosa cells; T: theca cells; S: stromal cells). Representative IHC images of staining for total and phospho-YAP (objective 63×) and their antibody control (NEG) showing negative staining of total YAP and phospho-YAP (objective 63×). Structure and cellular architecture of both groups can be observed in the H&E representative images.

We next determined the cellular and subcellular localization of total and phosphorylated YAP (phospho-YAP at serine 127: pYAP; Ser 127) proteins in both non-cystic and cystic follicles (Figure 3). Immunostaining for total YAP was observed in the nuclei and cytoplasm of GCs and TCs in both cystic and non-cystic follicles. A more intense staining detected in nuclei and cytoplasm of both cell layers from the cyst group (in comparison to respective cell layers and subcellular compartments in control group) suggested a higher expression of total YAP protein in cystic follicles. Phospho-YAP staining was also observed in the cytoplasm and nuclei of granulosa cell layers in both cyst and control follicles. 

Interestingly, GCs from cystic follicles seemed to present not only a lower number of nuclei positive for phospho-YAP, but also the intensity of such a signal in the nucleus and in the cytoplasm of these cells was weaker when compared to respective cells from the control group. Most interestingly, while the theca cell layer from cyst follicles presented some extent of positive signal for phospho-YAP (predominantly in their cytoplasm), a poor to absent signal for phospho-YAP was detected in theca cells from non-cystic control follicles.

### 3.3. Expression Levels for YAP, TEADs and Classic YAP-TEAD Target Genes in Cystic Granulosa Cells 

**Figure 4:**
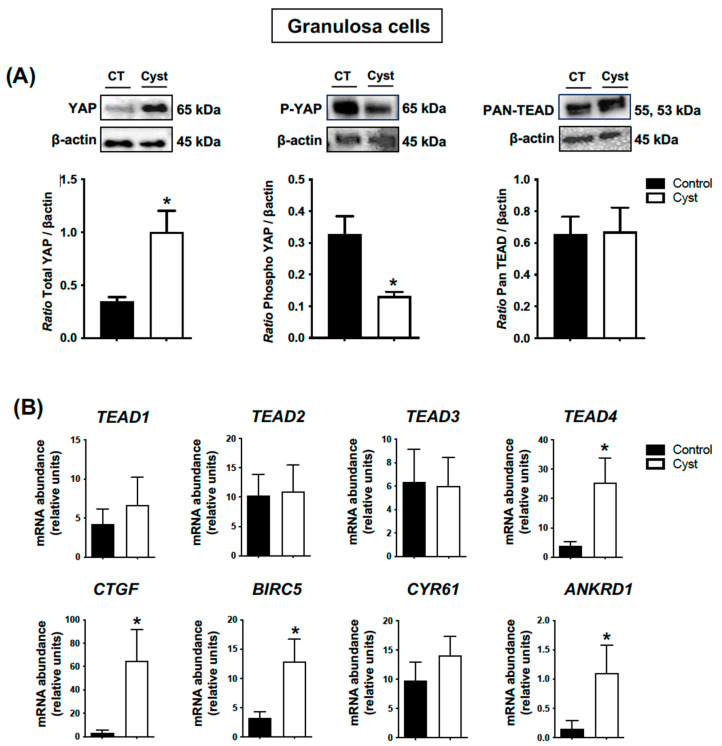
**Expression levels for YAP (total and phospho forms), TEADs and classic YAP-TEAD target genes in cystic granulosa cells.** Granulosa cells were isolated from ovarian follicular cysts and from non-cystic large control follicles. Total and phospho-YAP (pYAP; Ser 127) and Pan-TEAD protein levels (**A**) were measured by Western blot (WB) and normalized to β-actin (n = 5 for each group). Relative messenger RNA abundance (**B**) was measured by real-time qPCR and normalized to the geometric mean of three housekeeping genes *H2AFZ*, *GAPDH* and *RLP19* (n = 10 for each group). Data represent the group mean ± SEM. An asterisk (*) indicates difference between groups (*p* < 0.05).

To complement our IHC analyses and to better assess if YAP total protein levels were indeed augmented in GCs isolated from cyst group samples, we then employed Western blot analyses. The results demonstrated that total YAP protein levels were significantly higher in GCs from cystic follicles in comparison to GCs from control follicles, but also that phospho-YAP abundance was decreased (*p* < 0.05; Figure 4A) whereas total TEAD protein levels (Pan-TEAD) were not different. 

We then measured the abundance of mRNA encoding for *TEAD 1–4* and the classic YAP-TEAD target genes connective tissue growth factor (*CTGF*), baculoviral IAP repeat-containing protein 5 (*BIRC5*), cysteine-rich angiogenic inducer 61 (*CYR61*) and ankyrin repeat domain 1 (*ANKRD1)* as a measure of Hippo pathway activity. The results showed that the mRNA abundance for *TEAD4* was significantly increased in the cyst group (*p* < 0.05; Figure 4B) while the other TEAD family members (*TEAD1*, *TEAD 2* and *TEAD3*) were not statistically different between the control and cyst group (*p* > 0.05; Figure 4B), and that *CTGF*, *BIRC5* and *ANKRD1* mRNA abundance was significantly higher in cyst group in comparison to controls (*p* < 0.05; Figure 4B). Together, these findings showed that YAP total expression is augmented in cystic GCs as phospho-YAP protein levels decrease and, consequently, its TEAD-related transcriptional activity increases. 

### 3.4. Expression Levels for YAP, TEADs and Classic YAP-TEAD Target Genes in Cystic Theca Cells

**Figure 5:**
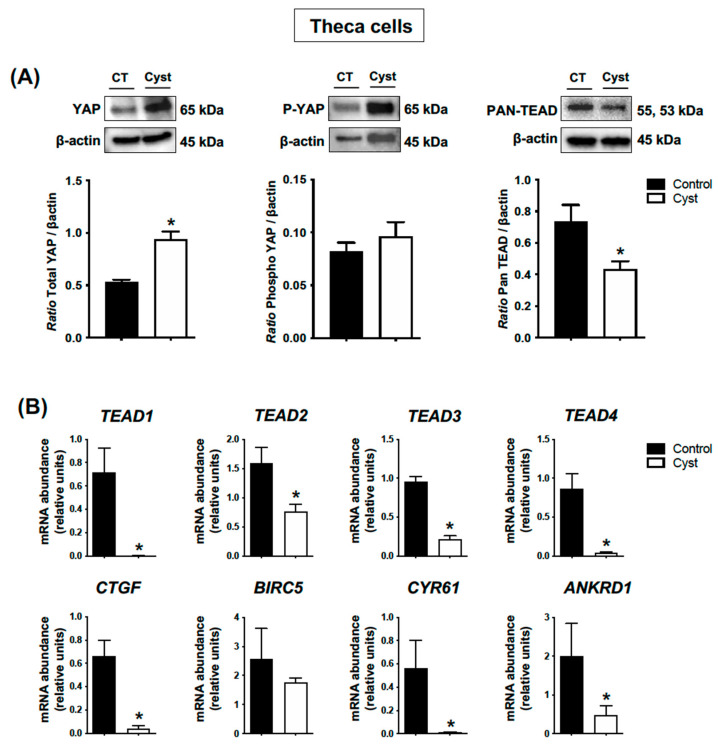
**Expression levels for YAP (total and phospho forms), TEADs and classic YAP-TEAD target genes in cystic theca cells.** Theca cells were isolated from ovarian follicular cysts and from non-cystic large control follicles. Total and phospho-YAP (pYAP; Ser 127) and Pan-TEAD protein levels (**A**) were measured by Western blot (WB) and normalized to β-actin (n = 4 for each group). Relative messenger RNA abundance (**B**) was measured by real-time qPCR and normalized to the geometric mean of three housekeeping genes *H2AFZ*, *GAPDH* and *RLP19* (n = 4 for each group). Data represent the group mean ± SEM. An asterisk (*) indicates difference between groups (*p* < 0.05).

In contrast to the results suggested by the IHC analyses, the results obtained with Western blot assessment did not confirm a significant increase in phospho-YAP protein levels in theca cells from cystic follicles when compared to controls (*p* > 0.05; Figure 5A). On the other hand, total YAP protein levels were significantly increased in cystic follicles (*p* < 0.05; Figure 5A). Pan-TEAD immunoreactive bands indicated lower TEAD protein expression levels in theca cells from cystic follicles compared with control follicles (*p* < 0.05; Figure 5A), and correspondingly the abundance of mRNA encoding *TEAD1*, *TEAD2*, *TEAD*3 and *TEAD4* were all significantly lower in TCs from cysts in comparison with TCs from control follicles (*p* < 0.05; Figure 5B). The abundance of mRNA encoding classic YAP-TEAD target genes *CTGF*, *CYR61* and *ANKRD1* were also significantly lower in TCs from cysts in comparison to TCs from control follicles (*p* < 0.05; Figure 5B). Together, these results indicated that the YAP-TEAD-related transcriptional activity in TCs from cysts differs to what was observed in granulosa cells.

## 4. Discussion

In the past 50 years, many studies have clarified important aspects of cystic ovarian disease (COD) characterization in cattle; nevertheless, the core players that orchestrate the alterations occurring in the large antral follicle and that culminate with cyst formation and persistence remain uncertain. In the present study, we used spontaneous ovarian follicular cysts to show the first evidence that the deregulation of Hippo effector YAP expression and/or activity can be a potential key to better understand COD pathogenesis. We show herein that YAP expression and transcriptional activity is significantly increased in granulosa cells of cystic follicles in comparison to that observed in non-cystic large preovulatory follicles. Conversely, YAP-TEAD-related transcriptional activity in theca cells from cysts is highly compromised, most likely due to lower expression levels for TEADs observed in these cells.

Phenotypically, bovine ovarian cysts are normally defined as anovulatory ovarian structures with a cavity greater than 20 mm in diameter and that persist for at least 10 days in the absence of a functional corpus luteum. The main macroscopic difference between follicular cysts and luteinized follicular cysts is based on their follicle wall thickness which is less than 3 mm in follicular cysts and greater than 3 mm in luteinized follicular cysts [26,27,28]. The diagnosis of COD due to the presence of a follicular anovulatory cyst is, however, significantly more frequent in dairy farms [29]. In addition to macroscopic and histological evaluations, we also validated the follicular cyst-like status of our samples performing molecular and hormone analyses which were compatible with several previous reports. As expected for GCs [22,30,31], the mRNA abundance for the classic overexpressed COD markers *PGRb*, *AR* and *STAR* was significantly increased in our cyst groups in comparison to controls. In addition, a known characteristics of COD follicles is their low responsiveness to gonadotropins due to decreased gonadotropin receptors in GCs [30,31,32,33]. Indeed, mRNA levels for both *LHCGR* and *FSHR* were significantly lower in our cyst group samples in comparison to controls. 

Although the experimental samples analyzed herein do not allow us to state conclusively that YAP expression and transcriptional activity has a causal effect on COD pathogenesis, the potential functions exerted by CTGF, BIRC5 and ANKRD1 (YAP-TEAD-target genes that were increased in cystic GCs herein) may represent strong evidence of its potential key role in cyst formation and persistence. In addition to the lower expression of gonadotropin receptors described previously, GCs in bovine ovarian cysts generally have low rates of proliferation and of apoptosis [34,35]. Interestingly, the conditional deletion of *Ctgf* in mouse GCs increases *Lhcgr* mRNA levels and concomitantly increases GCs apoptosis [36]. In addition, recombinant CTGF protein suppresses proliferation of the human SVOG granulosa cell line [37]. BIRC5 protein is associated with both inhibition of apoptosis and regulation of the cell cycle in chicken granulosa cells. It can interact with components of the G2/M checkpoint and preserve the integrity of the cellular mitotic-related apparatus as it may act as an anti-apoptotic protein by attenuating caspase-3 activity [38]. Finally, ANKRD1 has been shown to induce gene expression of the well-known anti-apoptotic marker BCL2 [39]. Taken together, the functions normally exerted by these altered classic YAP-TEAD genes can be directly related with the main pillars established so far for COD functional pathogenesis, which include altered patterns of differentiation, proliferation and apoptosis in cystic GCs.

The main GCs-related results showed herein are compatible with important findings in humans. First, YAP was identified as a susceptibility gene for polycystic ovarian syndrome (PCOS) in humans [18,40,41], and second it has been suggested that increased YAP mRNA and protein levels in GCs from PCOS patients accelerate the evolution of PCOS [39,42]. Both of the latter studies employed associative approaches (methylation status of YAP promoter activity and genome-wide associations with PCOS, respectively) and therefore did not test experimentally a causal effect of YAP deregulation on the formation of the ovarian cysts in women. Although we still need to perform further studies to confirm such causal effect in cattle too, the results obtained with the spontaneously occurring cystic follicles employed herein show us strong evidence that YAP-TEAD-dependent transcriptional activity can be indeed directly related to the functional and morphological changes normally observed in bovine follicular cysts. Therefore, as for humans, YAP-TEAD signaling components may represent an important potential therapeutic target for the treatment of COD in cattle. The potential success of this type of treatment will not only be extremely important for the dairy industry, as it may represent an alternative model to translate treatments into women, but bovine models of ovarian cyst studies could also be used to test drugs and protocols that may be useful for humans affected by PCOS. Women and cows actually present common aspects at the ovarian physiology level. The sizes of follicles at distinct stages of development and the dynamics of follicle wave emergence and growth are similar in both species [42,43,44,45,46]. In addition, cattle and women share many features related to reproductive aging [47].

A puzzling finding of our study is the paradox between YAP expression vs. YAP-TEAD transcriptional activity in theca cells from cystic follicles. In these cells, total YAP protein abundance increased with no change in phospho-YAP, suggesting an accumulation of non-phosphorylated YAP, and it is this unphosphorylated YAP that can accumulate in the nucleus and activate TEAD-dependent transcription [9]. However, abundance of classic TEAD-response genes (*CTGF*, *CYR61* and *ANKRD1)* decreased in TCs from cystic follicles. One explanation is the decrease in TEAD expression observed in these cells, such that even in the presence of increased YAP the availability of TEAD proteins became a limiting factor and abrogated expression of the Hippo target genes. However, the mechanism behind such a decrease and its relevance for COD pathogenesis remains to be elucidated. 

## 5. Conclusions

We provide a novel insight that the Hippo pathway effector YAP may play a key role in the pathogenesis of cystic ovarian disease in dairy cattle. Further studies are still required to establish its causal effect in the etiology of COD, and to determine the factors/conditions leading to YAP deregulation in ovarian follicle cells. Nonetheless, this first evidence points to YAP as a potential therapeutic target for the treatment of this major ovarian disorder in cattle.

## Figures and Tables

**Table 1:** List of antibodies used for IHC and WB.

Name of Antibody	Manufacturer (cat. no.)	Type	Dilution WB	Dilution IHC
ß-actin (C4)	Santa Cruz (sc-47778 HRP)	CkM	1:10,000	
YAP (D8H1X)	Cell signaling (14074)	RbM	1:1000	1:250
Phospho-YAP (Ser127) (D9W2I)	Cell signaling (13008)	RbM	1:1000	1:250
Pan-TEAD (D3F7L)	Cell signaling (13295)	RbM	1:1000	
Anti-Rabbit IgG-HRP Conjugate	Promega (W401B)	Rb	1:1000	

CkM: Chicken monoclonal; RbM: rabbit monoclonal; Rb: rabbit.

**Table 2:** Sequences of primers used in the expression analysis of target genes.

Gene	Sequence 5′→3′	Accession Number
*ANKRD1*	F: ATCAGTGCGCGGGATAAGTT	NM_001034378.2
	R: GGGAGTATCTCCTTCCCGGT	
*AR*	F: CCTGGTTTTCAATGAGTACCGCATG	[22]
	R: TTGATTTTTCAGCCCATCCACTGGA	
*BIRC5*	F: CTGAGAACGAGCCCGACTTG	NM_001001855.3
	R: ATGTTCTTCTATAGGGTCGTCATCT	
*CTGF*	F: AGCTGAGCGAGTTGTGTACC	[15]
	R: TCCGAAAATGTAGGGGGCAC	
*CYP19A1*	F: CTGAAGCAACAGGAGTCCTAAATGTACA	[23]
	R: AATGAGGGGCCCAATTCCCAGA	
*CYR61*	F: GGCTCCCCGTTTTGGAATG	NM_001034340.2
	R: TCATTGGTAACGCGTGTGGA	
*ESR1*	F: AGGGAAGCTCCTATTTGCTCC	[22]
	R: CGGTGGATGTGGTCCTTCTCT	
*ESR2*	F: CTTCGTGGAGCTCAGCCTGT	NM_174051
	R: GAGATATTCTTTGTGTTGGAGTTT	
*FSHR*	F: AGCCCCTTGTCACAACTCTATGTC	NM_174061.1
	R: GTTCCTCACCGTGAGGTAGATGT	
*GAPDH*	F: GATTGTCAGCAATGCCTCCT	NM_001034034.2
	R: GGTCATAAGTCCCTCCACGA	
*H2AFZ*	F: GAGGAGCTGAACAAGCTGTTG	[23]
	R: TTGTGGTGGCTCTCAGTCTTC	
*LHCGR*	F: TGGCTGGGATTATGACTATGGTT	[24]
	R: ATTTCCCGTGATGGCTAGGATA	
*PGRb*	F: TGCGAGACCCCCAGAGAAGGA	XM_583951
	R: GCGCCAGCAGGGTGTCCAG	
*RLP19*	F: CCGGCTGCTTAGACGATACC	NM_001040516.1
	R: CCGCTTGTTTTTGAACACGTT	
*STAR*	F: CCCAGCAGAAGGGTGTCATC	[24]
	R: TGCGAGAGGACCTGGTTGAT	
*TEAD1*	F: CACAAGACGTCAAGCCCTTTG	[15]
	R: CCAGGCGAAGTTTGGTTGTG	
*TEAD2*	F: CTGAATGTGGACCAGGTTTCCAA	[15]
	R: GACCAAAACTGGAAAAGCTCCG	
*TEAD3*	F: CGAATATTCCGCCTTCATGGAG	[15]
	R: TATCTGACGCACATCCACTGC	
*TEAD4*	F: ATGTCGTCCGCCCAGATCAT	[15]
	R: AGGTTTCACATCATGGGACGTT	

Forward (F) and reverse (R) primers used in RT-qPCR.

## Data Availability

Not applicable.
